# How Did the COVID-19 Lockdown Affect Children and Adolescent's Well-Being: Spanish Parents, Children, and Adolescents Respond

**DOI:** 10.3389/fpubh.2021.746052

**Published:** 2021-11-25

**Authors:** Sara Ajanovic, Jon Garrido-Aguirre, Bàrbara Baro, Núria Balanza, Rosauro Varo, Pere Millat-Martínez, Sara Arias, Jordi Fonollosa, Alexandre Perera-Lluna, Iolanda Jordan, Carmen Muñoz-Almagro, Elisenda Bonet-Carne, Aina Crosas-Soler, Esther Via, Begonya Nafria, Juan José García-García, Quique Bassat

**Affiliations:** ^1^ISGlobal, Hospital Clínic—Universitat de Barcelona, Barcelona, Spain; ^2^Institut de Recerca Sant Joan de Déu, University of Barcelona, Barcelona, Spain; ^3^Universitat Politècnica de Catalunya, BarcelonaTech, Barcelona, Spain; ^4^B2SLab, Departament d'Enginyeria de Sistemes, Automàtica i Informàtica Industrial, Universitat Politècnica de Catalunya, Barcelona, Spain; ^5^Centro de Investigação em Saúde de Manhiça, Maputo, Mozambique; ^6^Networking Biomedical Research Centre in the Subject Area of Bioengineering, Biomaterials and Nanomedicine (CIBER-BBN), Madrid, Spain; ^7^Institut de Recerca Sant Joan de Déu, Barcelona, Spain; ^8^Pediatric Intensive Care Unit, Hospital Sant Joan de Déu, University of Barcelona, Barcelona, Spain; ^9^Consorcio de Investigación Biomédica en Red de Epidemiología y Salud Pública, Madrid, Spain; ^10^Department of Medicine, Universitat Internacional de Catalunya, Barcelona, Spain; ^11^Molecular Microbiology Department, Hospital Sant Joan de Deu, Esplugues, Barcelona, Spain; ^12^BCNatal Fetal Medicine Research Center (Hospital Clínic and Hospital Sant Joan de Déu), University of Barcelona, Barcelona, Spain; ^13^Institut d'Investigacions Biomèdiques August Pi i Sunyer, Barcelona, Spain; ^14^Child and Adolescent Psychiatry and Psychology Department, Hospital Sant Joan de Déu, Barcelona, Spain; ^15^Pediatrics Department, Hospital Sant Joan de Déu, Universitat de Barcelona, Barcelona, Spain; ^16^ICREA, Catalan Institution for Research and Advanced Studies, Barcelona, Spain

**Keywords:** COVID-19, lockdown, children, adolescent, mental health, well-being

## Abstract

**Background:** During the COVID-19 pandemic, lockdown strategies have been widely used to contain SARS-CoV-2 virus spread. Children and adolescents are especially vulnerable to suffering psychological effects as result of such measures. In Spain, children were enforced to a strict home lockdown for 42 days during the first wave. Here, we studied the effects of lockdown in children and adolescents through an online questionnaire.

**Methods:** A cross-sectional study was conducted in Spain using an open online survey from July (after the lockdown resulting from the first pandemic wave) to November 2020 (second wave). We included families with children under 16 years-old living in Spain. Parents answered a survey regarding the lockdown effects on their children and were instructed to invite their children from 7 to 16 years-old (mandatory scholar age in Spain) to respond a specific set of questions. Answers were collected through an application programming interface system, and data analysis was performed using R.

**Results:** We included 1,957 families who completed the questionnaires, covering a total of 3,347 children. The specific children's questionnaire was completed by 167 kids (7–11 years-old), and 100 adolescents (12–16 years-old). Children, in general, showed high resilience and capability to adapt to new situations. Sleeping problems were reported in more than half of the children (54%) and adolescents (59%), and these were strongly associated with less time doing sports and spending more than 5 h per day using electronic devices. Parents perceived their children to gain weight (41%), be more irritable and anxious (63%) and sadder (46%). Parents and children differed significantly when evaluating children's sleeping disturbances.

**Conclusions:** Enforced lockdown measures and isolation can have a negative impact on children and adolescent's mental health and well-being. In future waves of the current pandemic, or in the light of potential epidemics of new emerging infections, lockdown measures targeting children, and adolescents should be reconsidered taking into account their infectiousness potential and their age-specific needs, especially to facilitate physical activity and to limit time spent on electronic devices.

## Background

Nearly 80–90% of school-age youth could not physically attend school in more than 160 countries during the first wave of the coronavirus disease 2019 (COVID-19) pandemic (1, 2). In Spain, one of the first measures implemented when cases started increasing was to close all schools and impose a strict home confinement. During the first period of the lockdown (March 14th–April 27th of 2020), referred as “strict lockdown” hereinafter, only essential activities were allowed, and children were compelled to stay home except for emergency situations. From April 28th to June 21st, children were progressively allowed to leave the household in a very controlled manner and for a limited period (i.e., 1 h per day with no close interactions), during what became referred as the “relaxed lockdown.” Schools remained closed and pupils attended online lectures whenever possible. Children did not return to face-to-face learning until September 2020. Home confinement measures due to the COVID-19 pandemic, which were particularly stringent for children, may have had deleterious effects on the physical and mental health of this particularly vulnerable age group (3, 4). Herein, we aimed to understand the perceived impact of lockdown measures on the mental health and well-being of minors, as reported by parents, but also adolescents and children themselves. We focused on risk perception and attitudes toward lockdown, perceptions of schooling, emotional responses, changes in biorhythms, psychical activity, sleep and eating attitudes, and screen time.

## Methods

### Study Design

We performed a cross-sectional study using convenience sampling through an online survey, launched in July 2020 and available until November 2020. The study was promoted *via* social media and the landing page of the Kids Corona Project. The questionnaire (see Supplementary Material) was created by a team of pediatricians at Hospital Sant Joan de Déu of Barcelona, Spain, and was available in Spanish or Catalan (the two official languages in Catalonia). Questions specifically referred to the strict lockdown but also enquired about feelings/perceptions at the moment of survey completion (which was after the lockdown). To avoid collecting disturbances present before lockdown, we asked about “new problems detected during lockdown.” Most questions had yes/no as possible answers, but some asked respondents to choose between few closed answers or grade a perception on a scale from 0 (minimum) to 10 (maximum). Families living in Spain with children under 16 years of age were invited to participate and to answer questions regarding COVID-19 and the physical, mental health and wellbeing of children during lockdown. Parents answered the survey regarding their children (0–16 years). Those children old enough to read and answer the questionnaire themselves, and who were in the age group for which there is mandatory schooling (>6–<16 years of age), were invited to answer the survey. Children aged 7–11 years were instructed to do it with caregivers' support, and adolescents over 11 years old were instructed to do it on their own.

### Data Collection

The answers to these surveys were collected through an API system using a custom code written in Python (5). Selection criteria for household enrollment were (i) accepted informed consent, (ii) respondent aged 18 years old or more, (iii) one or more children under 16 years old, and (iv) declared a valid Spanish postal code. Additionally, children and adolescents' answers were considered if both parents and the participating minor provided assent.

### Statistical Analysis

The analysis was performed using R language (6). The significance level for the statistical tests was α = 0.05; Bonferroni correction was applied when using multiple tests. Chi-squared test was applied to tabulated counts of categorical data (*p*-values are estimated using Monte Carlo method in the case of small cell counts). Mann–Whitney test is applied to numerical answers; the reported *p*-value results from the corresponding one-sided test.

### Ethical Considerations

The study was conducted in accordance with the protocol and the Declaration of Helsinki (current version Fortaleza, Brazil, October 2013), and following the relevant legal requirements (Law 14/2007 of July 3, 2007, on Biomedical Research). The study protocol, informed consent forms and data collection tools were approved by the Ethics Committee for clinical research of the Hospital Sant Joan de Déu, Barcelona, Spain (C.I. PIC-123-20), prior to study initiation.

## Results

### Demographics

A total of 2,054 families answered the survey, of which 1,957 met the study inclusion criteria. The included surveys covered a total of 3,347 children: 1,463 aged 0–6 years, 1,284 aged 7–11 years, and 600 adolescents aged 12–15 years. All parents answered a questionnaire, one for each daughter or son, independently. Additionally, 13% of the older children (167 answers) and 17% of the adolescents (100 answers) completed the questionnaire specifically addressed to their respective age group. In 57% of the households there was at least 1 adult working in a highly COVID-19 exposed environment (e.g., health centers, hospitals, or old people's retirement homes). The main participants sociodemographic characteristics are presented in Table 1.

**Table 1:** Characteristics of the 1,957 families included in the study.

**VARIABLE**	
**Age of parents (median, IQR) (years)**	42 [IQR: 38–46]
**Regions**	
Barcelona	1,328 (67.86%)
Madrid	154 (7.87%)
Tarragona	96 (4.91%)
Girona	95 (4.85%)
Lleida	53 (2.71%)
Zaragoza	33 (1.69%)
Alicante	19 (0.97%)
Valencia	18 (0.92%)
Asturias	12 (0.61%)
Illes Balears	12 (0.61%)
Others^*^	137 (7.00%)
**Children per household**	
1	743 (37.97%)
2	1,046 (53.45%)
3	147 (7.51%)
4	14 (0.71%)
5 or more	7 (0.36%)
**Children age**	
0–6	1,463 (43.71%)
7–11	1,284 (38.36%)
12–15	600 (17.93%)
**Adult working in a highly exposed COVID-19 environment**	
Yes	1,110 (56.72%)
No	847 (43.28%)
**Households reporting one confirmed SARS-CoV-2 infection**	120 (6.13%)
**Households reporting one suspected SARS-CoV-2 infection**	239 (12.21%)
**Number of rooms in household**	
1	10 (0.51%)
2	251 (12.83%)
3	998 (51.00%)
4	570 (29.13%)
5 or more	128 (6.54%)
**Number of bathrooms in household**	
1	520 (26.57%)
2	1,151 (58.82%)
3	250 (12.77%)
4 or more	36 (1.84%)
**Number of cohabitant elderly people**	
0	1,812 (92.59%)
1	70 (3.58%)
2 or more	75 (3.83%)

* *Given that the questionnaire was available online, we received responses from a variety of Spanish regions outside of Catalonia. Out of Spain were excluded (see inclusion criteria). Regions with <10 respondents have been grouped as “others”*.

### Risk Perception and Transmission Knowledge

At the time of survey completion, 6% of the households reported at least one family member having been affected or currently infected by the severe acute respiratory syndrome coronavirus 2 (SARS-CoV-2) (microbiologically confirmed infection), and 12% reported at least one suspected case. The majority (84%) of families knew someone infected and 20% knew someone close who had died of COVID-19. Among families with either a suspected or microbiologically confirmed infection, 47% considered a 5–15 year-old child to have been the index case, 22% a 0–4 year-old, 12% a >16 year-old, and 19% reported a simultaneous infection among more than one household member (of varying ages). Median time to subsequent secondary cases was 6.5 days (interquartile range [IQR]: 3–10). When a case was confirmed or suspected at home, measures adopted varied widely between families: 42% followed a strict lockdown of the family, 21% a strict lockdown of the affected person, 16% used a facemask at home, 19% ensured an exclusive use of the sleeping room and bathroom, 19% restricted the use of common spaces, and 39% did not follow any of the aforementioned measures. Regarding parents' opinion about children's role in the pandemic, 42% perceived them to be at high risk of getting infected by SARS-CoV-2, 50% thought strict lockdown measures for children were necessary, 60% considered that children should have had permission to go outside in a controlled way since the first day, and 65% found re-opening of schools was appropriate (98% of them suggested to do it with safety measures in place). During strict lockdown, 88% of children never set foot on the street, while during relaxed lockdown the majority (54%) spent time outdoors at least 3–6 days a week for <1 h/day. Masks were worn by 95% of older children and adolescents and by almost half of the children below 6 years of age, even if this was not mandatory in Spain.

### Coping With Confinement

**Figure 1:**
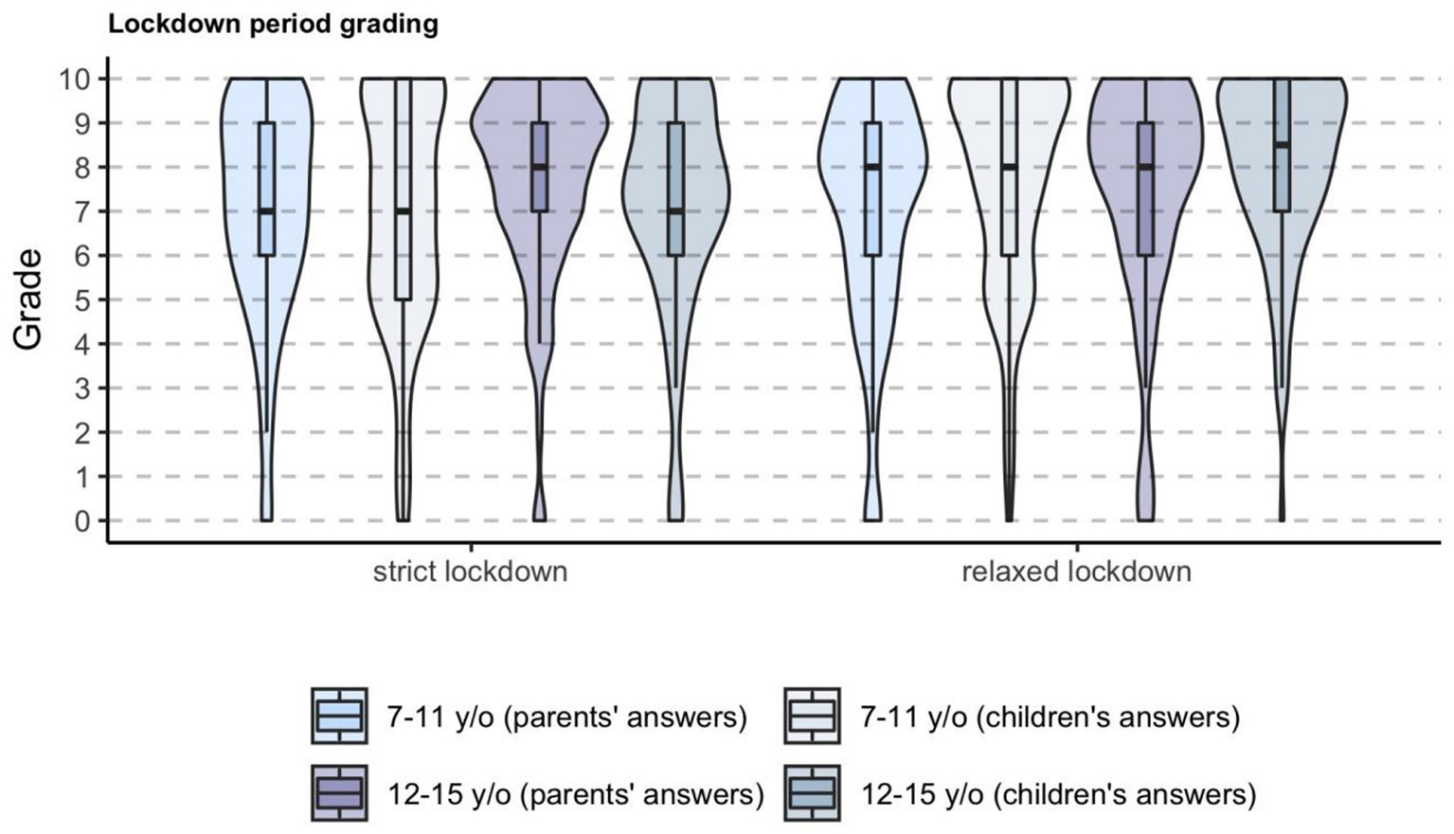
Overall coping with strict and relaxed lockdown according to parents and to children and adolescents.

The overall rate of good coping with confinement (rated on a 0–10 scale) was generally very positive. Parents scored their children an 8 [IQR: 6–9] both during strict and relaxed lockdown. Older children scored themselves a 7 [IQR: 5–10] and an 8 [IQR: 6–10], and adolescents scored themselves a 7 [IQR: 6–9] and an 8.5 [IQR: 7–10], during strict and relaxed lockdown, respectively (Figure 1). When asking if they would accept another strict lockdown if necessary due to a hypothetical worsening of the pandemic, 73% of parents, 81% of older children, and 83% of adolescents would accept it. Of note, we found no differences in these answers between children living with a confirmed/suspected case of SARS-CoV-2 and those living without it. One in every five families had an episode of a child needing healthcare attention during strict lockdown (58% febrile episodes, 4% acute mental health problems).

### Biorhythms (Physical Activity, Alimentary Patterns, and Sleeping Disturbances)

**Figure 2:**
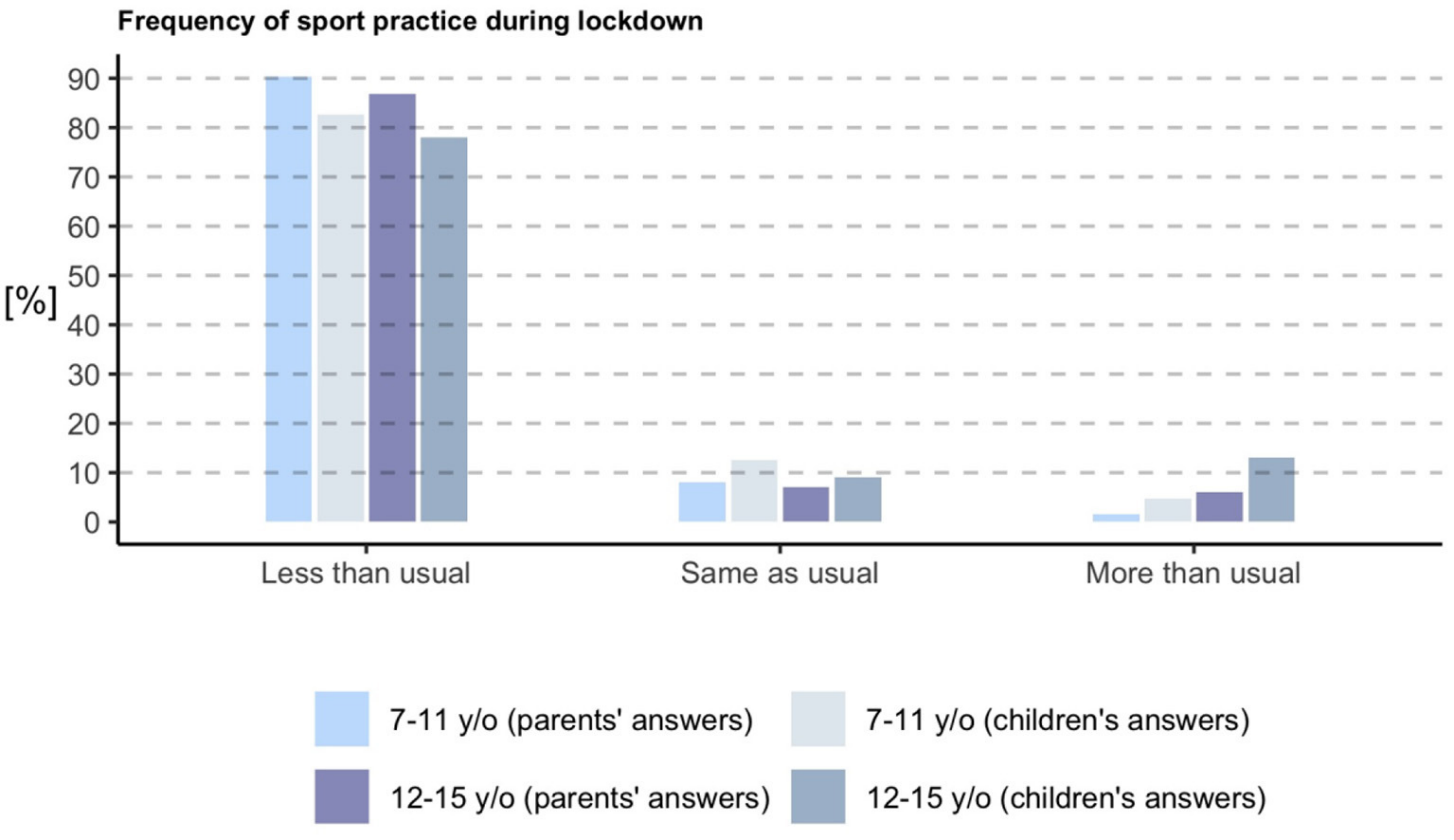
Time spent doing sports according to parents and to children and adolescents. According to parents' perception, during strict lockdown 90% of older children and 81% of adolescents practiced less sports than usual, similarly as children themselves reported when asked.

According to parents' perception, during strict lockdown 90% of older children and 81% of adolescents practiced less sports than usual (Figure 2), and 64% of older children and 39% adolescents did not do any kind of sports. On the other hand, during relaxed lockdown, 65% of children and adolescents used the allowed time outdoors to do some kind of sport. When asking parents about weight and eating disturbances, 10% perceived in their children to lose weight and 41% to gain weight. Additionally, 17% reported new eating problems not present before lockdown such as eating less, more and/or in different periodicity than usual, being picky with food, and eating due to anxiety or boredom.

**Figure 3:**
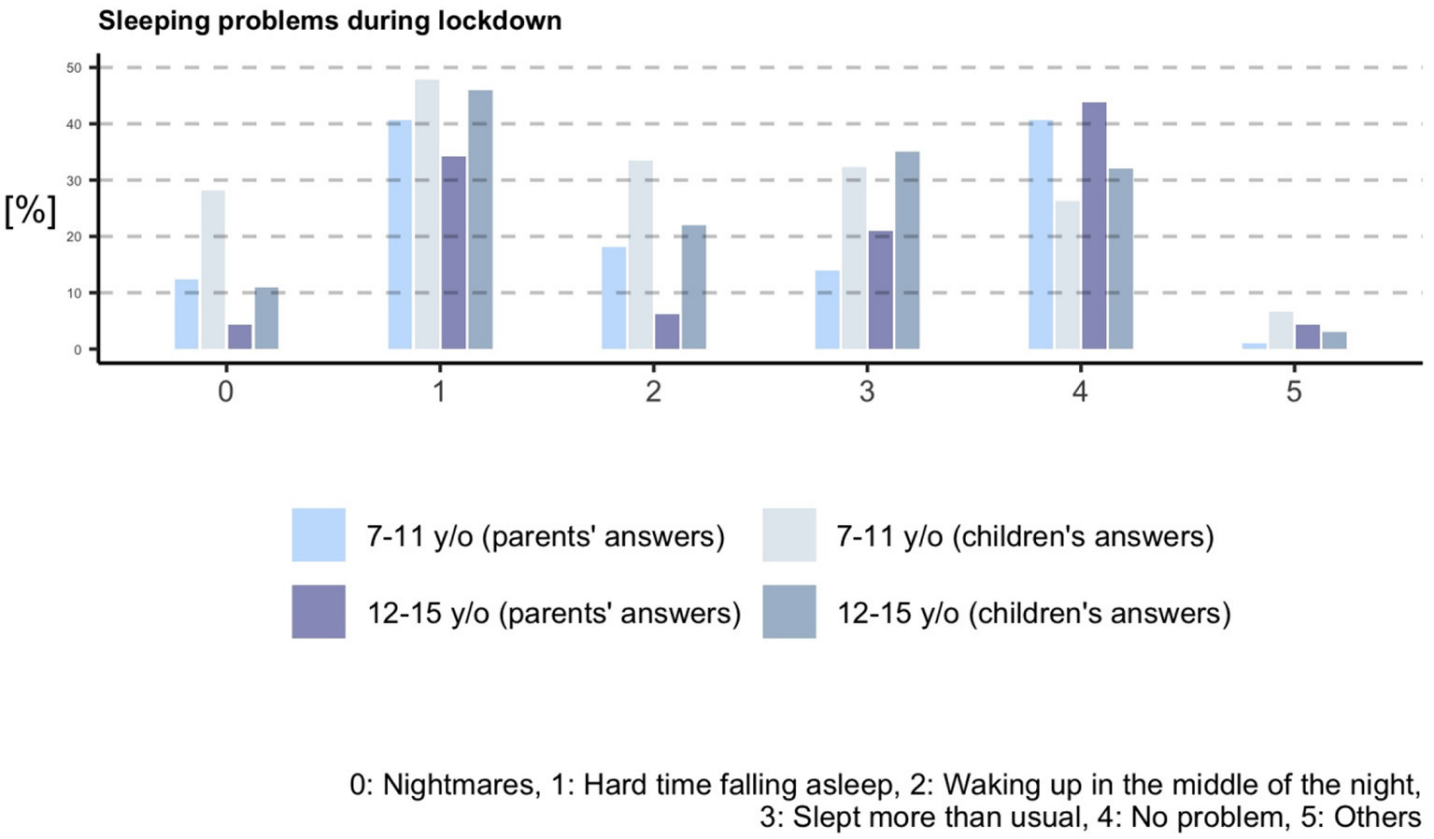
Sleeping problems during lockdown according to parents and to children and adolescents.

Parents reported sleeping problems during the strict lockdown in 54% of young children, 57% of older children and 59% of adolescents. The most common problem among all age groups was hard time falling asleep. Interestingly, when children and adolescents answered, they reported more sleeping disturbances than their parents (*p* = 0.000004 and *p* = 0.009). In older children, higher self-perception was observed for awakening in the middle of the night (Parents Answering [PA]: 18%, Children Answering [CA]: 33%), sleeping more than usual (PA: 14%, CA: 32%), having a hard time falling asleep (PA: 40%, CA: 48%), and having nightmares (PA: 12%, CA: 28%). Similarly, increased self-perception in adolescents was observed for awakening in the middle of the night (PA: 6%, CA: 22%), sleeping more than usual (PA: 21%, CA: 35%), having a hard time falling asleep (PA: 34%, CA: 46%), and having nightmares (PA: 4%, CA: 11%; Figure 3).

### Emotional Distress

When we asked parents about emotional distress signs observed in their children, 63% reported to have witnessed them to be more anxious than usual, 63% to be more irritable, 46% to be sadder, and 34% to have other emotional alterations. More than half of older children reported themselves to be more anxious (62%), irritable (55%), and sadder (52%), with median intensities from 4 to 5 out of 10 during strict lockdown. In adolescents, these feelings were reported in 49, 46, and 41% individuals, respectively, with median intensities from 3 to 5 out of 10 during strict lockdown.

### Electronic Devices and Social Media

Parents reported that at least one type of electronic device (i.e., smartphone, television or online audiovisual content, videogames, or social media) was used more than usual during strict lockdown in 77% of younger children, 93% of older children, and 95% of adolescents. The most widely used device was television together with online audiovisual content, with 75% of younger children, 85% of older children, and 82% of adolescents using it more often than before the lockdown. Playing videogames also increased in 65% of older children and 62% of adolescents. In addition, adolescents also increased use of their smartphone (68%) and social media (61%). The use time of electronic devices increased with age, being 1–3 h/day the predominant option among all children and 3–5 h/day the predominant option among adolescents. Higher use of social media increased during lockdown in comparison with the usual utilization before lockdown, and was self-reported by 28% of older children and 52% of adolescents. These results are summarized in Table 2.

**Table 2:** Answers about sleeping troubles, sports, and electronic devices use reported by parents for each child, distributed by age groups.

	**Age group**					
	**0–6**		**7–11**		**12–15**	
	***N***	**%**	***N***	**%**	***N***	**%**
	1,463		1,284		600	
**During strict lockdown, has your child experienced**						
Trouble falling asleep?	523	35.7%	522	40.7%	248	41.3%
Sleeping more than usual?	91	6.2%	149	11.6%	116	19.3%
Having nightmares?	220	15.0%	176	13.7%	23	3.8%
Awakening in the middle of the night?	327	22.4%	202	15.7%	50	8.3%
**During strict lockdown has your child practiced any sports?**						
Yes	596	40.7%	694	54.0%	368	61.3%
No	867	59.3%	590	46.0%	232	38.7%
**During strict lockdown, how many hours has your child used electronic**						
**devices (excepting schooling hours)?**						
Not daily	303	20.7%	30	2.3%	4	0.7%
Less than 1 h per day	187	12.8%	107	8.3%	19	3.2%
1–3 h per day	649	44.4%	652	50.8%	159	26.5%
3–5 h per day	258	17.6%	365	28.4%	203	33.8%
More than 5 h per day	66	4.5%	130	10.1%	215	35.8%
**Sports activity during strict lockdown**						
Less than usual	1,064	72.7%	1,149	89.5%	485	80.8%
Same as usual	339	23.2%	79	6.2%	54	9.0%
More than usual	60	4.1%	56	4.4%	61	10.2%
**Videogames time during strict lockdown**						
Less than usual	196	13.4%	71	5.5%	39	6.5%
Same as usual	901	61.6%	380	29.6%	188	31.3%
More than usual	366	25.0%	833	64.9%	373	62.2%
**Reading time during strict lockdown**						
Less than usual	240	16.4%	304	23.7%	153	25.5%
Same as usual	844	57.7%	588	45.8%	315	52.5%
More than usual	379	25.9%	392	30.5%	132	22.0%
**Time spent listening to music during lockdown**						
Less than usual	115	7.9%	96	7.5%	40	6.7%
Same as usual	715	48.9%	710	55.3%	282	47.0%
More than usual	633	43.3%	478	37.2%	278	46.3%
**Smartphone time during strict lockdown**						
Less than usual	143	9.8%	102	7.9%	27	4.5%
Same as usual	899	61.4%	637	49.6%	162	27.0%
More than usual	421	28.8%	545	42.4%	411	68.5%
**Online/TV audiovisual content (television, online content, etc.)**						
**during strict lockdown**						
Less than usual	50	3.4%	15	1.2%	12	2.0%
Same as usual	321	21.9%	174	13.6%	96	16.0%
More than usual	1,092	74.6%	1,095	85.3%	492	82.0%
**Time spent on social media during strict lockdown**						
Less than usual	231	15.8%	136	10.6%	24	4.0%
Same as usual	1,150	78.6%	781	60.8%	211	35.2%
More than usual	82	5.6%	367	28.6%	365	60.8%
**Playing (handicrafts, boardgames, etc.) during strict lockdown**						
Less than usual	103	7.0%	62	4.8%	73	12.2%
Same as usual	282	19.3%	261	20.3%	278	46.3%
More than usual	1,078	73.7%	961	74.8%	249	41.5%
**Has the child worn a mask when going outdoors?**						
Yes	730	49.9%	1,204	93.8%	574	95.7%
No	733	50.1%	80	6.2%	26	4.3%

### Schooling

Parents scored satisfaction with remote schooling in a 0–10 scale, with a median of 6 [IQR: 4–9] for younger children, and with a median of 7 [IQR: 5–8] for older children. Children themselves scored it with a median of 7 [IQR: 5–8]. The most common score was 10 among the three groups. Both parents of adolescents and teenagers themselves scored schooling satisfaction with a median of 7 [IQR: 5–9], but in this group the mode score was 10 for parents and 8 for the students. When children were asked if they were satisfied with school activities, 26% of children and 35% of adolescents were not. Importantly, 81% of children reported to feel comfortable with schools re-opening compared to a 65% of adolescents, and 52% of the latter thought it was necessary.

### Comparisons and Inference

In all age groups, we found higher number of reported sleeping problems (either by parents or by children) among those knowing someone who died of COVID-19 compared to those who did not. Older children who reported to have felt fear or anxiety about going out reported a higher incidence of sleeping disturbances than the ones who did not feel that way (56 vs. 39.3%, *p* = 0.047). In general, sleeping problems were less likely to happen among children who did sport during lockdown vs. the ones who did not (49.15 vs. 41.67%), especially regarding having trouble falling asleep and sleeping more than usual. Doing sport activities as usual seems to be mildly protective against sleeping problems when compared to spending less time than usual (*p* = 0.04). Spending more than 5 h per day looking at screens was a risk factor for some of the sleeping disturbances in teenagers, when compared to 1–3 h of daily use (*p* = 0.00087).

Regarding the reported feelings by children and adolescents, the score on irritability was significantly lower among kids (5 [IQR: 2–7] vs. 6 [IQR: 3–8], *p* = 0.0062) and adolescents (4 [IQR: 1–6] vs. 6 [IQR: 3–7], *p* = 0.015) who did sport as compared to those who did not. In addition, less physical activity than usual was also found to be associated with an increased feeling of sadness in adolescents, as compared to spending more time than usual doing sport (*p* = 0.01). The increased use of social networks above usual levels was found to be associated with increased anxiety levels in teenager reported answers (*p* = 0.0077). Among adolescents, being unsatisfied with academic training during lockdown was found to be associated with anxiety (*p* = 0.04), and fear of going out in the street was associated with increased perceived sadness (*p* = 0.00017).

## Discussion

Here, we described how COVID-19 lockdown has affected children and adolescents' well-being using a set of online questionnaires targeting families in Catalonia and Spain, which reported the status of 3,347 children.

We found that, at the onset of the pandemic, parental perception of children's risk of infection and transmission were generally high, probably supported by the containment measures applied in many countries, which included schools' closure. In other studies, the greatest risk perception has been found in women with lower education who have children in their care (7). However, recent research suggests that children are less (8) or equally (9) likely to become infected than adults when exposed to a case and, when infected, children's symptoms are usually milder (9, 10). Interestingly, despite the high-risk initial perception, the majority of parents would have preferred their children to be allowed to spend time outdoors during the entire lockdown period and strongly supported the re-opening of schools with prevention measures in place. It has been recently demonstrated that children's role in SARS-CoV-2 transmission is likely to be weaker than for other age groups during school re-opening due to the prevention tools adopted and children compliance with them (11).

Parents reported children to cope with confinement reasonably well, according to a previous study where parents reported a more engagement than disengagement-oriented coping strategy in Spanish children during lockdown (12). Parents, but especially children and adolescents, reported to cope better during the relaxed lockdown compared to the strict lockdown. The change in lockdown measures was positively rated and the allowed time outdoors was mostly used for sports activities. Actually, it has been shown that children missed outdoor exercise during strict lockdown (13), and that having an outdoor exit in the house (e.g., garden, terrace) contributed to lower levels of psychological and behavioral symptomatology (14). Indeed, we showed physical and sports activities were highly decreased during lockdown in most children and adolescents as previously reported (13–16), and 39% of children and adolescent did not do sport at all. In addition, 41% of parents reported their children to have suffered weight increases, probably as a result of the reduced physical activity and the reduction of fruit and vegetable consumption (15).

We found sleeping disturbances during lockdown were reported for the majority of children and adolescents, in accordance with previous studies (14–17). Importantly, we observed that children and adolescents self-reported more sleeping disturbances than what was reported by their parents. Given many of these disturbances are probably not easily observed by caregivers (i.e., nightmares, awakening in the night) unless children are directly asked, interviewing children and adolescents about their well-being should be included in this type of studies, and children's participation in surveys should be further promoted. Despite clear instructions to parents to encourage their children and adolescents to respond a questionnaire, we obtained low participation of children and adolescents in our survey. Eventhough, we still reached a total of 267, allowing making valuable conclusions about their self-reported well-being.

On the other hand, knowing someone who died from COVID-19 was associated with sleeping disturbances, as well as the statement “being afraid or anxious about going outside.” Accordingly, it has been shown that children who knew someone who had suffered from COVID-19 at home or whose parent was directly involved in the pandemic, presented higher anxiety scores (18). Of note, anxiety and irritability were reported for the majority of children and adolescents, being the youngest the most affected. Similar results have been recently described (14, 17, 19, 20) and underline the need to consider how we communicate information to children and help them manage this information. Furthermore, it has been shown that anxiety and depressive symptoms and other negative outcomes were more likely in children whose parents reported higher levels of stress, depression or were unemployed (18, 20–23), and that those were often more important among the youngest ones (20). Thus, supporting parents' mental health and providing them with accurate information, strategies and support to cope with lockdown is essential to protect their children's mental health (24, 25).

We also found that sleeping disturbances were associated with doing less sports than usual, which supports allowing outdoor activity and sports during lockdown periods to protect children's mental health and well-being. Actually, it has been shown a significantly different sleeping time between strict and relaxed confinement (15). Of note, children who could go outside during COVID-19 restrictions, as well as children whose parents were less stressed, were more likely to meet the physical activity suggested by WHO guidelines (24), which in turn resulted in reduced psychosocial difficulties (16). The increase in anxiety, irritability, sadness and changes in biorhythms are adaptive symptoms to stressful situations, and although the expectation is that they will slowly improve when the pandemic restrictions are eased, it is possible that a number of these children and adolescents develop a more serious mental health problem.

In accordance with recent studies, we also found a dramatic increase in electronic devices' usage in all age groups (14, 15, 23, 26), which has been shown to pose a risk on mental health regardless of the pandemic. The use of Internet has undoubtedly facilitated to some extent the continuity of social and schooling activity during lockdown, especially during strict lockdown. However, deprivation of sports and outdoors activities in favor of screen time consumption might be a damaging trade-off. We found an association between spending more time in electronic devices (>5 h per day) and sleeping disturbances, as well as with reported irritability, sadness and anxiety. In line with our findings, frequent social media exposure has been shown to be positively associated with anxiety (26). Interestingly, lower screen exposure was observed for the relaxed lockdown period (15).

Interestingly, and despite having endured the strictest lockdown, most children and adolescents reported acceptance to go back to a strict lockdown if this was required due to the epidemiological situation, reflecting their high resilience and capability to adapt to new situations. The reported acceptance of prevention measures was very high, and even though the use of facemask was not among the most popular measures according to parents, older children and adolescents reported to wear it when spending time outdoors. Of note, the use of facemask is not mandatory in Spain under 6 years of age; however, its use was reported for half of this young children's age group. Despite the possible social desirability bias, the use of facemasks is certainly extended among children. Indeed, a recent study in Spain found that risk perception and age groups with the highest self-perceived risk of disease (51–64 years of age) were also those who went most often outside their homes (27, 28). This differs markedly from our results among children, who scrupulously followed the rules and would do so again. Furthermore, children whose parents were more resilient and adopted prevention and safety measures presented fewer negative responses during lockdown (12), pointing to the impact of adults adhering to prevention measures not only in terms of viral spread, but also in children's safety perception and emotional well-being.

A major strength of our study is the large number of families who answered the survey, largely superior to previous studies carried out in Spain and capturing information about 3,347 children. Another major strength is the collection of questionnaires directly from children and adolescents, which is not often included in similar studies. On the other hand, since our study is based on questionnaires answered by parents and their children after the lockdown periods studied, it could be affected by a recall bias and social desirability bias. However, the surveys performed to Spanish children and adolescents during the strict lockdown showed similar emotional and behavioral alterations (21, 24, 29). Another limitation is the timespan during which the questionnaires could be completed, which included few months and the beginning of the second COVID-19 wave in Spain, something that could have affected the responses. On the other hand, using online surveys based on volunteer participation probably resulted in a biased population, where underrepresentation of families with less-resources (which might lack a smartphone or computer, and/or lack access to the internet) is certainly present. This could have influenced the answers obtained, such as satisfaction with remote schooling, which was relatively good in our study. In addition, since our online survey did not include sensitive personal data, we could not exclude data from repetition of study participants. However, families were asked to answer the survey only once and data analysis suggests these instructions have been generally followed, given we found no repetition of descriptive family data among participants. Finally, since this is a cross-sectional study, we cannot infer cause-effect relationships. However, our associations could be further explored in future studies to determine if a cause-effect relationship exists. Although longitudinal assessments of the lockdown effects in children are needed, our results point out the importance of considering mental health and well-being protection during lockdown measures implementation to control COVID-19 outbreaks.

In summary, our study showed a negative impact of strict lockdown in the mental health and well-being of children and adolescents but also showed their high resilience and capability to adapt to new situations. Lockdown measures should consider their age-specific needs in future waves of the pandemic and in similar potential emergencies, facilitating physical activity and limiting time spent on electronic devices. Maintaining schools open or, at least, ensuring children can spend a minimum time outdoors, can help address these priorities.

## Data Availability Statement

The raw data supporting the conclusions of this article will be made available by the authors, without undue reservation.

## Ethics Statement

The study protocol, informed consent forms, and data collection tools were approved by the Ethics Committee for Clinical Research of the Hospital Sant Joan de Déu, Barcelona, Spain (C.I. PIC-123-20), prior to study initiation. Written informed consent to participate in this study was provided by the participants' legal guardian/next of kin.

## Author Contributions

QB, JG-G, SAj, BB, NB, RV, PM-M, and SAr: conceptualization and methodology. QB, AC-S, BN, and JG-A: survey preparation and software development. JG-A, JF, and AP-L: data collection, curation, data analysis, and statistics. SAj: writing-original draft. BB, NB, RV, PM-M, SAj, and JG-A: writing-review and editing. JG-G and QB: funding acquisition and project administration. All authors contributed to the article and approved the submitted version.

## Funding

This study was supported by Banco Santander and Stavros Niarchos Foundation, through KIDS corona platform. This study has been partially funded by Stavros Niarchos Foundation (SNF), Banco Santander and other private donors of Kidscorona. ISGlobal receives support from the Spanish Ministry of Science and Innovation through the Centro de Excelencia Severo Ochoa 2019–2023 Program (CEX2018-000806-S), and support from the Generalitat de Catalunya through the CERCA Program. This work was supported by the Spanish Ministry of Economy and Competitiveness (www.mineco.gob.es) TEC2014-60337-R, DPI2017-89827-R, Networking Biomedical Research Center in the subject area of Bioengineering, Biomaterials, and Nanomedicine (CIBER-BBN), initiatives of Instituto de Investigación Carlos III (ISCIII), and Share4Rare project (Grant Agreement 780262). B2SLab is certified as 2017 SGR 952. BB is a Beatriu de Pinós postdoctoral fellow granted by the Government of Catalonia's Secretariat for Universities and Research, and by Marie Sklodowska-Curie Actions COFUND Programme (BP3, 801370). NB was supported by an FPU predoctoral fellowship from the Spanish Ministry of Universities (FPU18/04260). JF acknowledges the support from the Serra Húnter program.

## Conflict of Interest

The authors declare that the research was conducted in the absence of any commercial or financial relationships that could be construed as a potential conflict of interest.

## Publisher's Note

All claims expressed in this article are solely those of the authors and do not necessarily represent those of their affiliated organizations, or those of the publisher, the editors and the reviewers. Any product that may be evaluated in this article, or claim that may be made by its manufacturer, is not guaranteed or endorsed by the publisher.

## Abbreviations

CA: Children answering
COVID-19: Coronavirus disease 2019
IQR: Interquartile range
PA: Parents answering
SARS-CoV-2: severe acute respiratory syndrome coronavirus 2.
