# Supplementary material for: How Did the COVID-19 Lockdown Affect Children and Adolescent's Well-Being: Spanish Parents, Children, and Adolescents Respond
Source: Front Public Health. 2021 Nov 25;9:746052. doi: 10.3389/fpubh.2021.746052 (PMC8655116; doi:10.3389/fpubh.2021.746052)
Supplement: Supplementary file 1 [file Data_Sheet_1.DOCX]

**La GRAN enquesta de Sant Joan de Déu sobre COVID-19 i salut pediàtrica**

**Idea**: Els estudis de recerca encetats des de la plataforma KIDS-Corona a l’hospital Sant Joan de Deu donaran a mig i llarg termini moltes respostes a la gran majoria de preguntes de recerca plantejades pels investigadors en relació a la infecció i malaltia pel SARS-CoV-2 que afecta a la població pediàtrica, i les particularitats en aquest grup específic d’edat (menors de 16 anys). Tanmateix, existeix una forta pressió per generar coneixement a més curt termini que permeti millor adreçar molts del reptes que la COVID-19 està plantejant en la nostre societat en relació als nens. Les “opinions de experts” de moment són les que permeten generar guies i recomanacions per aquells temes on la recerca està treballant activament i on es podrà confirmar les actuals hipòtesis quan els estudis estiguin terminats. Com a estratègia complementària, des de l’hospital Sant Joan de Déu, es vol obrir una enquesta participativa online per captar la opinió de pares i nens sobre temes de ampla rellevància.

Aquesta és una enquesta dirigida a la població general. Tanmateix, ens interessa particularment que hi responguin també famílies on hi ha hagut casos de COVID confirmats o sospitats.

**Mètode**: Seria interessant garantir una altíssima participació, i per assolir això, hem pensat en una enquesta participativa online, dividida en un màxim de 3-4 mòduls (cada un d’ells independent) i que sigui curta. Volem assolir un numero elevat de respostes, més que entrar en grans detalls en cada una de les respostes. Volem obrir l’enquesta a pares i nens (adolescents i també nens més petits >7 anys). L’enquesta estaria disponible online (utilitzant la plataforma KIDS-Corona, i un software d’enquestes online) des d’ara i durant un mes. L’objectiu és involucrar a milers de participants, i per això és indispensable una eina àgil i un número de preguntes limitat i concret. Seria bo també, per evitar saturar la població, que fos una única enquesta però que traslladi les necessitats de diferents grups del hospital, i per això es proposa un disseny via “mòduls” independents. Els mòduls que proposem d’entrada serien:

1. Covid-19 i nens: Preguntes sobre la transmissió, la prevenció, i la malaltia (dirigit a pares)
2. Confinament i salut física, mental i emocional dels menors
   1. Versió de l’enquesta per pares
   2. Versió de l’enquesta per menors adolescents (12-16a)
   3. Versió de l’enquesta per menors no adolescents >7a-11a
3. Mesures de desconfinament i tornada a activitats normals dels menors (inclosa la re-escolarització)
   1. Versió de l’enquesta per pares
   2. Versió de l’enquesta per menors adolescents (12-16a)
   3. Versió de l’enquesta per menors no adolescents >7a-11a

Lo ideal és que cada participant només passi un màxim de 5-10 minuts per mòdul (màxim de 15-20 preguntes per mòdul, amb preguntes tancades (estil “múltiple choice”) i espai final per comentaris. L’anàlisi de les respostes ens donarà una idea del pensament actual en relació a COVID-19 i salut dels nens, així com de la opinió de la societat (nens inclosos) sobre el procés de desconfinament i les recomanacions específiques pels nens. També ens hauria de permetre detectar patrons que recomanin la posada en marxa d’enquestes més específiques de cribratge de ansietat i trastorns emocionals que apareguin a curt o llarg termini. L’enquesta, si s’aconsegueix que es respongui de forma massiva, ens donarà dades crues de forma ràpida sobre temes actualment molt “candents” tals i com la percepció de risc en la salut dels nens, les conseqüències negatives que el confinament pugui haver causat, o la opinió sobre la tornada a les escoles, les colònies d’estiu etc.

**Pregunta d’entrada:**

**Per favor, respongui una única vegada a aquesta enquesta**

**Pregunta 1**- **Edat del adult responent**

|__|__|anys

Codi postal del domicili familiar

|  |  |  |  |  |
| --- | --- | --- | --- | --- |

Ha signat el consentiment informat?

1. Sí
2. No

A més de la seva participació, té algun fill (>7anys i <16 anys) que voldria que respongui?

1. Sí
2. No

Dona vostè el consentiment informat per la seva participació?

1. Sí
2. No

Segons resposta a aquesta pregunta obrir la possibilitat de participar en:

- Adults: Qüestionaris 1, 2a i 3a
- 12-16anys: Qüestionaris 2b, 3b (necessita assentiment addicional per part dels menors)
- <12 anys: Qüestionaris 2c, 3c

**Mòdul 1:** Covid-19 i nens: Preguntes sobre la transmissió, la prevenció, i la malaltia (dirigit a pares)

**Pregunta 1**- Des de l’inici de l’any, hi ha hagut a casa algun cas de COVID-19?

1. Sí, amb confirmació diagnòstica per un test diagnòstic
   1. Si sí, especificar edat de cas 1
   2. Si sí, especificar edat de cas 2
   3. Si sí, especificar edat de cas ...
2. Creiem que sí, tot i que no tenim confirmació per test diagnòstic
   1. Si sí, especificar edat de cas 1
   2. Si sí, especificar edat de cas 2
   3. Si sí, especificar edat de cas ...
3. No ho creiem

(si la resposta a la pregunta prèvia és 1 o 2, obrir aquesta pregunta)

**Sub-Pregunta 1-1**- En cas d’haver patit un o més casos de COVID-19 (confirmats o sospitats) a casa, qui creieu que es va posar primer malalt?

1. Primer es va posar malalt un nen menor de 5 anys
2. Primer es va posar malalt un nen de entre 5 i 15 anys
3. Primer es va posar malalt un nen de més de 15 anys o un adult
4. Es van produir els casos al mateix temps mes o menys

**Sub-Pregunta 1-2**- En cas d’haver patit un o més casos de COVID-19 (confirmats o sospitats) a casa, quines d’aquestes mesures vàreu implementar a casa vostre?

A. Confinament estricte del cas afectat 1. Si; 2. No

B. Confinament estricte de tota la unitat familiar 1. Si; 2. No

C. Ús de mascareta al domicili 1. Si; 2. No

D. Ús d’habitació i bany exclusiu per l’afectat 1. Si; 2. No

E. Restricció de la utilització d’espais comuns per l’afectat 1. Si; 2. No

**Sub-pregunta 1-3**-En cas d’haver se produït algun cas secundari, quants dies després del cas primari va ocórrer?

Cas secundari 1: |__|__| dies

Cas secundari 2: |__|__| dies

Cas secundari 3: |__|__| dies

...

**Pregunta 2**- Coneixen a alguna persona que hagi patit COVID-19?

1. Sí
2. No

**Pregunta 3**- Coneixen a algun nen <15 anys que hagi patit COVID-19?

1. Sí, amb confirmació diagnòstica per un test diagnòstic
   1. Si sí, especificar edat de cas 1
   2. Si sí, especificar edat de cas 2
   3. Si sí, especificar edat de cas ...
2. Creiem que sí, tot i que no tenim confirmació per test diagnòstic
   1. Si sí, especificar edat de cas 1
   2. Si sí, especificar edat de cas 2
   3. Si sí, especificar edat de cas ...
3. No

**Pregunta 4**- Alguna persona del seu entorn proper ha mort per COVID-19 (o amb sospita de)

1. Sí
2. No

**Pregunta 5**- Quantes persones conviuen en el domicili familiar?

|__|__|

**Pregunta 6**- Quants menors de 16 anys (fins a 15 anys inclosos) viuen al domicili? |__|__|

(i especificar edats)

Edat menor 1|__|__|

Edat menor 2|__|__|

...

**Pregunta 7**- Quantes habitacions té el domicili on viu? |__|__|

**Pregunta 8**- Quants banys hi ha al domicili on viu? |__|__|

**Pregunta 9**- Comparteixen el(s) menor(s) habitació amb alguna persona?

1. Sí
2. No

Si si, donar detalls

**Pregunta 10**- Comparteixen el(s) menor(s) bany amb alguna persona?

1. Sí
2. No

**Pregunta 11**- Nº de cohabitants adults de 65 anys o més |__|__|

**Pregunta 12**- A la casa hi conviu algun animal?

1. Sí
2. No

Si sí, donar detalls

Gosso(s): |__|

Gat(s): |__|

Ocell(s): |__|

Hàmster(s) i altre(s) rosegador(s): |__|

Tortugues i rèptils: |__|

Altres : |__|

**Pregunta 13**- Algun dels menors va a la llar de infants?

1. Sí
2. No

**Pregunta 14**- Per anar a l’escola/llar d’infants, algun dels menors usa transport col·lectiu?

1. Sí
2. No

**Pregunta 15**- Algun dels menors té una malaltia crònica?

1. Sí
2. No

Si sí, donar detalls

**Pregunta 16**- Hi ha algun fumador a casa?

1. Sí
2. No

**Pregunta 17**- A la casa, algun dels adults té exposició a ambients amb alta ocupació (treball en hospitals, residencies, escoles, industria, restauració, locals d’oci, transport públic, biblioteques, etc.)

1. Sí
2. No

**Pregunta 18**- Feina dels adults de la casa

Adult 1: (Camp lliure)

Adult 2: (camp lliure)

**Pregunta 19**- Creu que els nens són una població vulnerable a emmalaltir pel COVID-19?

1. Sí
2. No

**Mòdul 2: 1. Confinament i salut física, mental i emocional dels menors**

(Versió de l’enquesta per pares)

**Pregunta 1**- Durant el confinament estricte a casa, algun dels nens ha estat més irritable de lo habitual?

1. Sí
2. No

**Pregunta 2**- Durant el confinament estricte a casa, algun dels nens ha estat més trist de lo habitual?

1. Sí
2. No

**Pregunta 3**- Durant el confinament estricte a casa, algun dels nens ha estat més ansiós de lo habitual?

1. Sí
2. No

**Pregunta 4**- Durant el confinament estricte a casa, algun dels nens ha tingut un episodi de malaltia que requerís una consulta mèdica, anar al centre de salut o a l’hospital?

1. Sí
2. No

Si si, donar detalls

**Pregunta 5**- Durant el confinament estricte a casa, algun dels nens ha tingut problemes per dormir?

1. Sí
2. No

Si si, marcar les que corresponguin (es pot marcar més de una):

Menor 1:

1. L’hi ha costat adormir-se

2. Ha dormit més de lo habitual

3. Ha tingut malsons

4. S’ha despertat enmig de la nit

5. Altres (especificar) _________________________________________

Menor 2:

1. L’hi ha costat adormir-se

2. Ha dormit més de lo habitual

3. Ha tingut malsons

4. S’ha despertat enmig de la nit

5. Altres (especificar) _________________________________________

Menor 3:

1. L’hi ha costat adormir-se

2. Ha dormit més de lo habitual

3. Ha tingut malsons

4. S’ha despertat enmig de la nit

5. Altres (especificar) _________________________________________

...

**Pregunta 6**- Durant el confinament estricte a casa, algun dels nens ha tingut problemes a l’hora de menjar (diferent del període pre-confinament)

1. Sí
2. No

Si sí, donar detalls

**Pregunta 7**- Durant el confinament estricte a casa, algun dels nens s’ha aprimat?

1. Sí
2. No

**Pregunta 8**- Durant el confinament estricte a casa, algun dels nens s’ha engreixat?

1. Sí
2. No

**Pregunta 9**- Durant el confinament estricte a casa, heu evidenciat qualsevol altre trastorn a alteració del estat emocional, psicològic o mental dels nens de la casa?

1. Sí
2. No

Si sí, donar detalls

**Pregunta 10**- Durant el confinament estricte a casa, el(s) nen(s) han pogut fer algun tipus de esport?

1. Sí
2. No

Si sí, donar detalls:

Menor 1: temps dedicat per dia |__|__| Hores

Menor 1: Tipus esport (camp obert)

Menor 2: temps dedicat per dia |__|__| Hores

Menor 2: Tipus esport (camp obert)

**Pregunta 11**- Quin temps han dedicat durant el confinament estricte a casa el(s) nen(s) a les pantalles? (sense comptar activitats relacionades amb escola online)

Menor 1

1. <1 hora al dia
2. 1-<3 hores al dia
3. 3-5 hores al dia
4. >5 hores al dia

Menor 2

1. <1 hora al dia
2. 1-<3 hores al dia
3. 3-5 hores al dia
4. >5 hores al dia

Menor 3

1. <1 hora al dia
2. 1-<3 hores al dia
3. 3-5 hores al dia
4. >5 hores al dia

...

**Pregunta 12**- Quina disponibilitat tenen a casa de ordinadors o tablets que puguin usar els nens per participar en les tasques educatives online?

1. Disposem de al menys un ordinador o tablet per ús individual de cada nen
2. Disposem de un ordenador o tablet que els nens poden usar de forma comunitària, per torns (diferent del dels adults)
3. Els únics ordinadors o tablets disponibles són els que utilitzem els adults, i hem hagut d’establir torns per que els poguessin usar
4. No disposem d’ordinador o tablets que puguin ser usats pels nens

**Pregunta 13**- Durant el confinament estricte a casa, ha dedicat més o menys temps a les següents activitats que de manera habitual?

**Menor 1**

**Esport:**

1. Menys temps del habitual; 2. Igual que sempre; 3. Més temps del habitual

**Videojocs:**

1. Menys temps del habitual; 2. Igual que sempre; 3. Més temps del habitual

**Lectura:**

1. Menys temps del habitual; 2. Igual que sempre; 3. Més temps del habitual

**Escoltar música:**

1. Menys temps del habitual; 2. Igual que sempre; 3. Més temps del habitual

**Ús de telèfon:**

1. Menys temps del habitual; 2. Igual que sempre; 3. Més temps del habitual

**Continguts audiovisuals online/televisió**

1. Menys temps del habitual; 2. Igual que sempre; 3. Més temps del habitual

**Xarxes socials:**

1. Menys temps del habitual; 2. Igual que sempre; 3. Més temps del habitual

**Temps de joc (Manualitats, jocs de taula etc.)**

1. Menys temps del habitual; 2. Igual que sempre; 3. Més temps del habitual

**Menor 2**

**Esport:**

1. Menys temps del habitual; 2. Igual que sempre; 3. Més temps del habitual

**Videojocs:**

1. Menys temps del habitual; 2. Igual que sempre; 3. Més temps del habitual

**Lectura:**

1. Menys temps del habitual; 2. Igual que sempre; 3. Més temps del habitual

**Escoltar música:**

1. Menys temps del habitual; 2. Igual que sempre; 3. Més temps del habitual

**Ús de telèfon:**

1. Menys temps del habitual; 2. Igual que sempre; 3. Més temps del habitual

**Continguts audiovisuals online/televisió**

1. Menys temps del habitual; 2. Igual que sempre; 3. Més temps del habitual

**Xarxes socials:**

1. Menys temps del habitual; 2. Igual que sempre; 3. Més temps del habitual

**Temps de joc (Manualitats, jocs de taula etc.)**

1. Menys temps del habitual; 2. Igual que sempre; 3. Més temps del habitual

**Menor 3**

**Esport:**

1. Menys temps del habitual; 2. Igual que sempre; 3. Més temps del habitual

**Videojocs:**

1. Menys temps del habitual; 2. Igual que sempre; 3. Més temps del habitual

**Lectura:**

1. Menys temps del habitual; 2. Igual que sempre; 3. Més temps del habitual

**Escoltar música:**

1. Menys temps del habitual; 2. Igual que sempre; 3. Més temps del habitual

**Ús de telèfon:**

1. Menys temps del habitual; 2. Igual que sempre; 3. Més temps del habitual

**Continguts audiovisuals online/televisió**

1. Menys temps del habitual; 2. Igual que sempre; 3. Més temps del habitual

**Xarxes socials:**

1. Menys temps del habitual; 2. Igual que sempre; 3. Més temps del habitual

**Temps de joc (Manualitats, jocs de taula etc.)**

1. Menys temps del habitual; 2. Igual que sempre; 3. Més temps del habitual

**…**

**Pregunta 14**- En una escala del 0-10 (0=Molt malament; 10=Perfectament) com qualificaria com els seus fills han aguantat el període de confinament estricte? (respongui per cada un dels seus fills)

Menor 1: |__|__|

Menor 2: |__|__|

Menor 3: |__|__|

**Pregunta 15**- En una escala del 0-10 (0=Molt malament; 10=Perfectament) com qualificaria com els seus fills han aguantat el període de confinament més relaxat? (des del dia que es va permetre la sortida dels nens una hora al dia)

(respongui per cada un dels seus fills)

Menor 1: |__|__|

Menor 2: |__|__|

Menor 3: |__|__|

**Pregunta 16**- Tipus de dieta dels nens:

Nen 1: Omnívora  Vegetariana  Vegana

Nen 2: Omnívora  Vegetariana  Vegana

Nen 3: Omnívora  Vegetariana  Vegana

**Pregunta 17**- Està satisfet/a de la formació acadèmica rebuda pels seus fills durant el període de confinament?

1. Sí
2. No

Per cada menor, valorar en una escala del 0-10 (0=Molt insatisfet, 10=totalment satisfet)

Menor 1: |__|__|

Menor 2: |__|__|

Menor 3: |__|__|

Si us plau, detalla les raons per la/les teva/es nota/es

**_______________________________________________________________________**

**Mòdul 2: 2. Confinament i salut física, mental i emocional dels menors**

(Versió de l’enquesta per menors adolescents 12-16anys)

**Pregunta 1**- Durant el confinament estricte a casa, t’has sentit més irritable de lo habitual?

1. Sí
2. No

Posa una nota a quant irritable t’has sentit (0=gens irritable; 10=molt irritable)

**Pregunta 2**- Durant el confinament estricte a casa, t’has sentit més trist de lo habitual?

1. Sí
2. No

Posa una nota a quant trist t’has sentit (0=gens trist; 10=molt trist)

**Pregunta 3**- Durant el confinament estricte a casa, t’has sentit més ansiós de lo habitual?

1. Sí
2. No

Posa una nota a quant ansiós t’has sentit (0=gens ansiós; 10=molt ansiós)

**Pregunta 4**- Durant el confinament estricte a casa, has tingut problemes per dormir?

1. Sí
2. No

Si sí, donar detalls

1. T’ha costat adormir-te

2. Has dormit més de lo habitual

3. Has tingut malsons

4. T’ha despertat enmig de la nit

5. Altres (especificar) _________________________________________

**Pregunta 5**- Durant el confinament estricte a casa, has tingut algun problema a l’hora de menjar

1. Sí
2. No

Si sí, donar detalls

**Pregunta 6**- Durant el confinament estricte a casa, t’has aprimat?

1. Sí
2. No

**Pregunta 7**- Durant el confinament estricte a casa, t’has engreixat?

1. Sí
2. No

**Pregunta 8**- Durant el confinament estricte a casa, has tingut qualsevol altre trastorn del estat emocional, psicològic o mental?

1. Sí
2. No

Si sí, donar detalls

**Pregunta 9**- Durant el confinament estricte a casa, has pogut fer algun tipus de esport?

1. Sí
2. No

Temps dedicat per dia |__|__| Hores

Tipus esport (camp obert)

**Pregunta 10**- Quin temps has dedicat durant el confinament estricte a casa a les pantalles? (sense comptar activitats relacionades amb escola online)

1. <1 hora al dia
2. 1-<3 hores al dia
3. 3-5 hores al dia
4. >5 hores al dia

**Pregunta 11**- Durant el confinament estricte a casa, has dedicat més o menys temps a les següents activitats que de manera habitual?

**Esport:**

1. Menys temps del habitual; 2. Igual que sempre; 3. Més temps del habitual

**Videojocs:**

1. Menys temps del habitual; 2. Igual que sempre; 3. Més temps del habitual

**Lectura:**

1. Menys temps del habitual; 2. Igual que sempre; 3. Més temps del habitual

**Escoltar música:**

1. Menys temps del habitual; 2. Igual que sempre; 3. Més temps del habitual

**Ús de telèfon:**

1. Menys temps del habitual; 2. Igual que sempre; 3. Més temps del habitual

**Continguts audiovisuals online/televisió**

1. Menys temps del habitual; 2. Igual que sempre; 3. Més temps del habitual

**Xarxes socials:**

1. Menys temps del habitual; 2. Igual que sempre; 3. Més temps del habitual

**Temps de joc (Manualitats, jocs de taula etc.)**

1. Menys temps del habitual; 2. Igual que sempre; 3. Més temps del habitual

**Pregunta 12**- En una escala del 0-10 (0=Molt malament; 10=Perfectament) com qualificaries que has aguantat el període de confinament estricte?

|__|__|

**Pregunta 13**- En una escala del 0-10 (0=Molt malament; 10=Perfectament) com qualificaries que has aguantat el període de confinament més relaxat?

|__|__|

**Pregunta 14**- Com definiries el teu tipus de dieta?

Omnívora  Vegetariana  Vegana

**Pregunta 15**- Estàs satisfet/a de la formació acadèmica rebuda durant el període de confinament?

1. Sí
2. No

Valora en una escala del 0-10 (0=Molt insatisfet, 10=totalment satisfet)

|__|__|

Si us plau, detalla les raons per la teva nota, i digues que t’agradaria que s’hagués fet diferent

**Pregunta 16**- Què és el que més t’ha agradat i el que menys durant el confinament?

Més:

Menys:

**_______________________________________________________________________**

**Mòdul 2: 3. Confinament i salut física, mental i emocional dels menors**

(Versió de l’enquesta per menors no adolescents 7-11a)

**Pregunta 1**- Durant el confinament estricte a casa, t’has sentit més irritable de lo habitual?

1. Sí
2. No

Posa una nota a quant irritable t’has sentit (0=gens irritable; 10=molt irritable)

**Pregunta 2**- Durant el confinament estricte a casa, t’has sentit més trist de lo habitual?

1. Sí
2. No

Posa una nota a quant trist t’has sentit (0=gens trist; 10=molt trist)

**Pregunta 3**- Durant el confinament estricte a casa, t’has sentit més ansiós de lo habitual?

1. Sí
2. No

Posa una nota a quant ansiós t’has sentit (0=gens ansiós; 10=molt ansiós)

**Pregunta 4**- Durant el confinament estricte a casa, has tingut problemes per dormir?

1. Sí
2. No

Si sí, donar detalls

1. T’ha costat adormir-te

2. Has dormit més de lo habitual

3. Has tingut malsons

4. T’ha despertat enmig de la nit

5. Altres (especificar) _________________________________________

**Pregunta 5**- Durant el confinament estricte a casa, has tingut algun problema a l’hora de menjar

1. Sí
2. No

Si sí, donar detalls

**Pregunta 6**- Durant el confinament estricte a casa, t’has aprimat?

1. Sí
2. No

**Pregunta 7**- Durant el confinament estricte a casa, t’has engreixat?

1. Sí
2. No

**Pregunta 8**- Durant el confinament estricte a casa, has tingut qualsevol altre trastorn del estat emocional, psicològic o mental?

1. Sí
2. No

Si sí, donar detalls

**Pregunta 9**- Durant el confinament estricte a casa, has pogut fer algun tipus de esport?

1. Sí
2. No

Temps dedicat per dia |__|__| Hores

Tipus esport (camp obert)

**Pregunta 10**- Quin temps has dedicat durant el confinament estricte a casa a les pantalles? (sense comptar activitats relacionades amb escola online)

1. <1 hora al dia
2. 1-<3 hores al dia
3. 3-5 hores al dia
4. >5 hores al dia

**Pregunta 11**- Durant el confinament estricte a casa, has dedicat més o menys temps a les següents activitats que de manera habitual?

**Esport:**

1. Menys temps del habitual; 2. Igual que sempre; 3. Més temps del habitual

**Videojocs:**

1. Menys temps del habitual; 2. Igual que sempre; 3. Més temps del habitual

**Lectura:**

1. Menys temps del habitual; 2. Igual que sempre; 3. Més temps del habitual

**Escoltar música:**

1. Menys temps del habitual; 2. Igual que sempre; 3. Més temps del habitual

**Ús de telèfon:**

1. Menys temps del habitual; 2. Igual que sempre; 3. Més temps del habitual

**Continguts audiovisuals online/televisió**

1. Menys temps del habitual; 2. Igual que sempre; 3. Més temps del habitual

**Xarxes socials:**

1. Menys temps del habitual; 2. Igual que sempre; 3. Més temps del habitual

**Temps de joc (Manualitats, jocs de taula etc.)**

1. Menys temps del habitual; 2. Igual que sempre; 3. Més temps del habitual

**Pregunta 12**- En una escala del 0-10 (0=Molt malament; 10=Perfectament) com qualificaries que has aguantat el període de confinament estricte?

|__|__|

**Pregunta 13**- En una escala del 0-10 (0=Molt malament; 10=Perfectament) com qualificaries que has aguantat el període de confinament més relaxat?

|__|__|

**Pregunta 14**- Estàs satisfet/a de els activitats de escola fetes durant el període de confinament?

1. Sí
2. No

Valora en una escala del 0-10 (0=Molt insatisfet, 10=totalment satisfet)

|__|__|

Si us plau, detalla les raons per la teva nota, i digues que t’agradaria que s’hagués fet diferent

**Pregunta 15**- Què és el que més t’ha agradat i el que menys durant el confinament?

Més:

Menys:

**Mòdul 3: 1. Mesures de desconfinament i tornada a activitats normals dels menors (inclosa la re-escolarització)**

(Versió de l’enquesta per pares)

**Pregunta 1**- Li sembla raonable que els nens no podessin sortir al carrer durant les primeres 5 setmanes de confinament?

1. Sí
2. No

**Pregunta 2**- Creu que els nens haurien hagut de poder sortir al carrer (de forma controlada) des del primer dia del confinament estricte?

1. Sí
2. No

**Pregunta 3**- Durant el confinament estricte, han sortit els seus nens en algun moment al carrer?

1. Sí
2. No

**Pregunta 4**- Durant el confinament més relaxat, han sortit els seus nens en algun moment al carrer?

1. Sí
2. No

Si sí, quants dies a la setmana: 1. Cap; 2. 1-2 dies/setmana; 3. 3-6 dies/setmana; 4. >=7

Si sí, quantes vegades al dia? 1. Una vegada; 2. Més d’una vegada

Si sí, quant temps aproximadament per sortida? 1. <1hora; 2 >=1 hora

**Pregunta 5**- En les sortides al carrer, han portat els seus nens mascareta?

1. Sí
2. No

Menor 1: Edat |__|__| Mascareta: 1. Si; 2. No

Menor 2: Edat |__|__| Mascareta: 1. Si; 2. No

Menor 3: Edat |__|__| Mascareta: 1. Si; 2. No

**Pregunta 6**- En les sortides al carrer, han fet algun tipus d’esport?

1. Sí
2. No

Si sí, donar detalls

**Pregunta 7**- Al tornar a casa després de les sortides al carrer, els nens s’han rentat les mans

1. Sí, sempre
2. Sí, de vegades
3. No

**Pregunta 8**- Al tornar a casa després de les sortides al carrer, ha dutxat/banyat als nens (o s’han dutxat/banyat?)

1. Sí
2. No

**Pregunta 9**- Utilitzen a casa els nens solucions/gels amb base hidroalcohòlica per higiene de mans?

1. Sí
2. No

**Pregunta 10**- Amb quina freqüència es renten les mans els nens a la casa cada dia?

Nen 1: >5 vegades  >3-<5 vegades  <3 vegades

Nen 2: >5 vegades  >3-<5 vegades  <3 vegades

Nen 3: >5 vegades  >3-<5 vegades  <3 vegades

**Pregunta 11**- Creus que les llars de infants/escoles/instituts haurien de reobrir coincidint amb la flexibilitat del confinament?

1. Sí
2. No

**Pregunta 12**- Si les llars de infants/escoles/instituts tornen a la seva activitat al setembre, creus que s’haurien d’assegurar mesures de protecció pels nens?

1. Sí
2. No

**Pregunta 13**- Si les llars de infants/escoles/instituts tornen a la seva activitat al setembre, et sentiries còmode enviant hi el(s) teu(s) nen(s)

1. Sí
2. No

**Pregunta 14**- En una escala del 0-10 (0=no és important; 10=és molt important), qualifica quan important consideres les mesures següents de protecció a les llars de infants/escoles/instituts

1. Escalonament horari d’entrada i sortida per grups d’edat |__|__|
2. Portar mascaretes durant les aules |__|__|
3. Portar mascaretes durant l’activitat esportiva |__|__|
4. Portar mascaretes durant el pati |__|__|
5. Portar guants |__|__|
6. Escalonar horaris de menjador |__|__|
7. Minimitzar reunions grupals/assemblees als centres educatius |__|__|
8. Oferir tests diagnòstics als alumnes |__|__|
9. Instal·lar depuradores d’aire/filtres |__|__|
10. Reduir el Nº de nens per classe a un màxim de 15 nens a les escoles |__|__|
11. Limitar i controlar el Nº nens en el transport escolar |__|__|
12. Organitzar diferents torns docents durant els dies/alternar dies de classes presencials |__|__|

**Pregunta 15**- En cas de rebrot de la transmissió, entendries la necessitat de tornar a confinar-nos a casa de forma estricte?

1. Sí
2. No

**_______________________________________________________________________**

**Mòdul 3:2. Mesures de desconfinament i tornada a activitats normals dels menors (inclosa la re-escolarització)**

(Versió de l’enquesta per menors adolescents 12-16anys)

**Pregunta 1**- Et sembla raonable que els menors de 15 anys no podessin sortir al carrer durant les primeres 5 setmanes de confinament?

1. Sí
2. No

**Pregunta 2**- Creus que els menors de 15 anys haurien hagut de poder sortir al carrer (de forma controlada) des del primer dia del confinament estricte?

1. Sí
2. No

**Pregunta 3**- Tenies ansietat/por per sortir al carrer?

1. Sí
2. No

**Pregunta 4**- En una escala del 0-10 (0=cap ganes; 10=moltíssimes ganes) com qualificaries les ganes que tenies de sortir al carrer?

|__|__|

**Pregunta 5**- En les sortides al carrer, has portat mascareta?

1. Sí
2. No

**Pregunta 6**- Creus que la mascareta és útil?

1. Sí
2. No

**Pregunta 7**- Utilitzes a casa solucions/gels amb base hidroalcohòlica per higiene de mans?

1. Sí
2. No

**Pregunta 8**- Amb quina freqüència et rentes les mans a casa cada dia?

>5 vegades  >3-<5 vegades  <3 vegades

**Pregunta 9**- Creus que les escoles/instituts haurien de reobrir coincidint amb la flexibilitat del confinament?

1. Sí
2. No

**Pregunta 10**- Si les escoles/instituts tornen a la seva activitat al setembre, et sentiries còmode tornant-hi?

1. Sí
2. No

**Pregunta 11**- En una escala del 0-10 (0=molt incòmode; 10=Molt còmode), qualifica quan còmode et sentiries davant la implementació de les mesures següents de protecció a les escoles/instituts

1. Escalonament horari d’entrada i sortida per grups d’edat |__|__|
2. Portar mascaretes durant les aules |__|__|
3. Portar mascaretes durant l’activitat esportiva |__|__|
4. Portar mascaretes durant el pati |__|__|
5. Portar guants |__|__|
6. Escalonar horaris de menjador |__|__|
7. Minimitzar reunions grupals/assemblees als centres educatius |__|__|
8. Oferir tests diagnòstics als alumnes |__|__|
9. instal·lar depuradores d’aire/filtres |__|__|
10. Reduir el Nº de nens per classe a un màxim de 15 nens a les escoles |__|__|
11. Limitar i controlar el Nº nens en el transport escolar |__|__|
12. Organitzar diferents torns docents durant els dies/alternar dies de classes presencials |__|__|

**Pregunta 12**- En cas de rebrot de la transmissió, entendries la necessitat de tornar a confinar-nos a casa de forma estricte?

1. Sí
2. No

**_______________________________________________________________________**

**Mòdul 3:3. Mesures de desconfinament i tornada a activitats normals dels menors (inclosa la re-escolarització)**

(Versió de l’enquesta per menors no adolescents 7-11a)

**Pregunta 1**- Tenies ansietat/por per sortir al carrer?

1. Sí
2. No

**Pregunta 2**- En una escala del 0-10 (0=cap ganes; 10=moltíssimes ganes) com qualificaries les ganes que tenies de sortir al carrer?

|__|__|

**Pregunta 3**- En les sortides al carrer, has portat mascareta?

1. Sí
2. No

**Pregunta 4**- Creus que la mascareta és útil?

1. Sí
2. No

**Pregunta 5**- Amb quina freqüència et rentes les mans a casa cada dia?

>5 vegades  >3-<5 vegades  <3 vegades

**Pregunta 6**- Si les escoles tornen a la seva activitat al setembre, et sentiries còmode tornant-hi?

1. Sí
2. No

**Pregunta 7**- Quan les escoles tornin a la seva activitat, et sentiries còmode portant mascareta a classe?

1. Sí
2. No

**Pregunta 8**- En cas de rebrot de la transmissió, entendries la necessitat de tornar a confinar-nos a casa de forma estricte?

1. Sí
2. No
